# Microbiota-derived butyrate suppresses group 3 innate lymphoid cells in terminal ileal Peyer’s patches

**DOI:** 10.1038/s41598-017-02729-6

**Published:** 2017-06-21

**Authors:** Sae-Hae Kim, Byeol-Hee Cho, Hiroshi Kiyono, Yong-Suk Jang

**Affiliations:** 1 Department of Molecular Biology and Institute for Molecular Biology and Genetics, Chonbuk National University, Jeonju, 54896 Korea; 2 Department of Bioactive Material Sciences and Research Center of Bioactive Materials, Chonbuk National University, Jeonju, 54896 Korea; 3 Division of Mucosal Immunology and International Research and Development Center for Mucosal Vaccines, Institute of Medical Science, The University of Tokyo, Tokyo, 108-8639 Japan

## Abstract

The regional specialization of intestinal immune cells is affected by the longitudinal heterogeneity of environmental factors. Although the distribution of group 3 innate lymphoid cells (ILC3s) is well characterized in the lamina propria, it is poorly defined in Peyer’s patches (PPs) along the intestine. Given that PP ILC3s are closely associated with mucosal immune regulation, it is important to characterize the regulatory mechanism of ILC3s. Here, we found that terminal ileal PPs of specific pathogen-free (SPF) mice have fewer NKp46^+^ ILC3s than jejunal PPs, while there was no difference in NKp46^+^ ILC3 numbers between terminal ileal and jejunal PPs in antibiotics (ABX)-treated mice. We also found that butyrate levels in the terminal ileal PPs of SPF mice were higher than those in the jejunal PPs of SPF mice and terminal ileal PPs of ABX-treated mice. The reduced number of NKp46^+^ ILC3s in terminal ileal PPs resulted in a decrease in *Csf2* expression and, in turn, resulted in reduced regulatory T cells and enhanced antigen-specific T-cell proliferation. Thus, we suggest that NKp46^+^ ILC3s are negatively regulated by microbiota-derived butyrate in terminal ileal PPs and the reduced ILC3 frequency is closely associated with antigen-specific immune induction in terminal ileal PPs.

## Introduction

The gastrointestinal tract has a unique immune system that is specialized to the gut microenvironment^1^. Although it forms a continuous tube, the small intestine can be divided into three main anatomical compartments: the duodenum, followed by the jejunum, and then the ileum^2^. The commensal microbes are one of the distinct factors differentially distributed within each anatomical compartment of the small intestine^3^. Interestingly, the terminal ileum, located anatomically in the small intestine, contains a microbiota similar to that of the colon, because it is close to the cecum and proximal colonic tissue^4^. For example, while *Lactobacillus*, *Streptococcus*, and *Enterococcus* are localized primarily in the jejunum, segmented filamentous bacteria, Enterobacteriaceae, *Bacteroides*, and *Clostridium* are found mainly in the ileum and proximal colon^5^.

This longitudinal heterogeneity of microbes in the intestinal tract gives rise to regional specialization in the intestinal microenvironment, through the differential distribution of metabolites, such that aryl hydrocarbon receptor (AHR) ligands and short-chain fatty acids (SCFAs) are present decreasingly and increasingly, respectively, upon descending through the intestinal tract^6, 7^. More importantly, recent studies have shown that these metabolites play immune modulatory roles. The best known examples are the AHR ligands, tryptophan metabolites produced by *Lactobacillus* spp., which modulate the activation of Th17 cells and group 3 innate lymphoid cells (ILC3s) in the small intestine^8, 9^. Short-chain fatty acids, such as butyrate, acetate, and propionate, are generated by colonic bacteria, including *Clostridium* spp. and *Alcaligenes* spp., and play roles as immune modulators of macrophages, dendritic cells (DCs), and regulatory T cells (Tregs)^10–12^. Butyrate, in particular, not only promotes the acetylation of histone H3 at lysine 27 at the Foxp3 promoter in naïve CD4^+^ T cells and induced Tregs, but also regulates antigen (Ag)-presenting cells through G protein-coupled receptor (GPR)109a-mediated signaling in the colon^12, 13^. However, the regulatory role of butyrate on ILCs has not been defined yet in the small intestine or in Peyer’s patches (PPs).

PPs are major immune inductive sites and well-characterized sites in terms of the induction of IgA responses to T cell-dependent Ags in the gut because PP-deficient mice have been shown to fail to develop Ag-specific IgA responses to particle forms of Ags^14^. The density of PPs increases from the jejunum to the ileum in the human small intestine and PP cells in mice consist of 60% B220^+^ B cells, 25% CD3^+^ T cells, and 10% CD11c^+^ DCs and other cells^15^. PPs form a distinct environment relative to lymph nodes, such that they always contain germinal centers due to the presence of M cells, which are specialized epithelial cells for the transport of luminal Ags which include microbes^16^. Importantly, luminal bacteria that exist differentially along the intestine also affect the construction of distinguishable cell populations^17^. In particular, there is a high frequency of Tregs in the lamina propria (LP) of the colon compared with the small intestine^18^. These differential distribution characteristics of the cells in the intestine are correlated with the density of *Clostridium* spp., which are localized in the colon and can drive the development of Tregs in the colon^19^. However, the distinctive features of and difference between the PPs of the jejunum and the ileum are still poorly understood in terms of immune cell populations and their biological roles in regulating the mucosal immune response.

ILCs are the most recently described family of lymphoid cells and have been categorized into three subsets, based on cytokine-secreting profiles and their specific transcription factor expression^20^. Group 1 innate lymphoid cells (ILC1s) express the transcription factor T-bet, primarily produce IFN-γ, and are related to immune responses to bacteria and protozoan parasites^21^. Group 2 innate lymphoid cells (ILC2s) express GATA3, produce IL-4, IL-5, IL-9, and IL-13, and contribute to the protection against helminth infection^21^. Finally, ILC3s express retinoid-related orphan receptor γt (RORγt) and produce IL-17 A, IL-22, lymphotoxin, and CSF2^22^. Owing to the heterogeneity of ILC3s, they are subdivided into three groups^23^. LTi-like CCR6-expressing ILC3s regulate the development of lymphoid cells and intestinal inflammation^24^. The T-bet^+^ natural cytotoxicity receptor NKp46^+^ and NKp46^−^ ILC3s contribute to the maintenance of tissue homeostasis, antibacterial responses, and autoimmune inflammation^25^. Although ILCs are present in small numbers, their role on mucosal surfaces is closely associated with the host defense against various infections through rapid activation of host-derived cytokine expression^26^. ILCs were initially assumed to play a major role in innate immunity, but recent studies suggested roles in adaptive immunity, including in Treg expansion and in the enhancement of IgA isotype switching^26–28^. In particular, PP ILC3s were shown to inhibit the proliferation of *Alcaligenes* spp.-specific CD4^+^ T cells in terminal ileal PPs and this inhibition was closely associated with the control of systemic inflammation induced by microbiota^29^. These findings suggest regulatory roles of ILCs in connecting and regulating innate and adaptive immune responses in PPs, although the regulatory mechanism of ILCs in PPs has not yet been clearly defined. In this study, we sought to understand the regulatory role of butyrate on ILC3s in PPs, especially in relation to the anatomical differential distribution of ILC3s in jejunal and terminal ileal PPs and its biological consequences in mucosal immune regulation.

## Results

### The RORγt^+^ ILC3s are distributed differentially in jejunal and terminal ileal PPs

**Figure 1:**
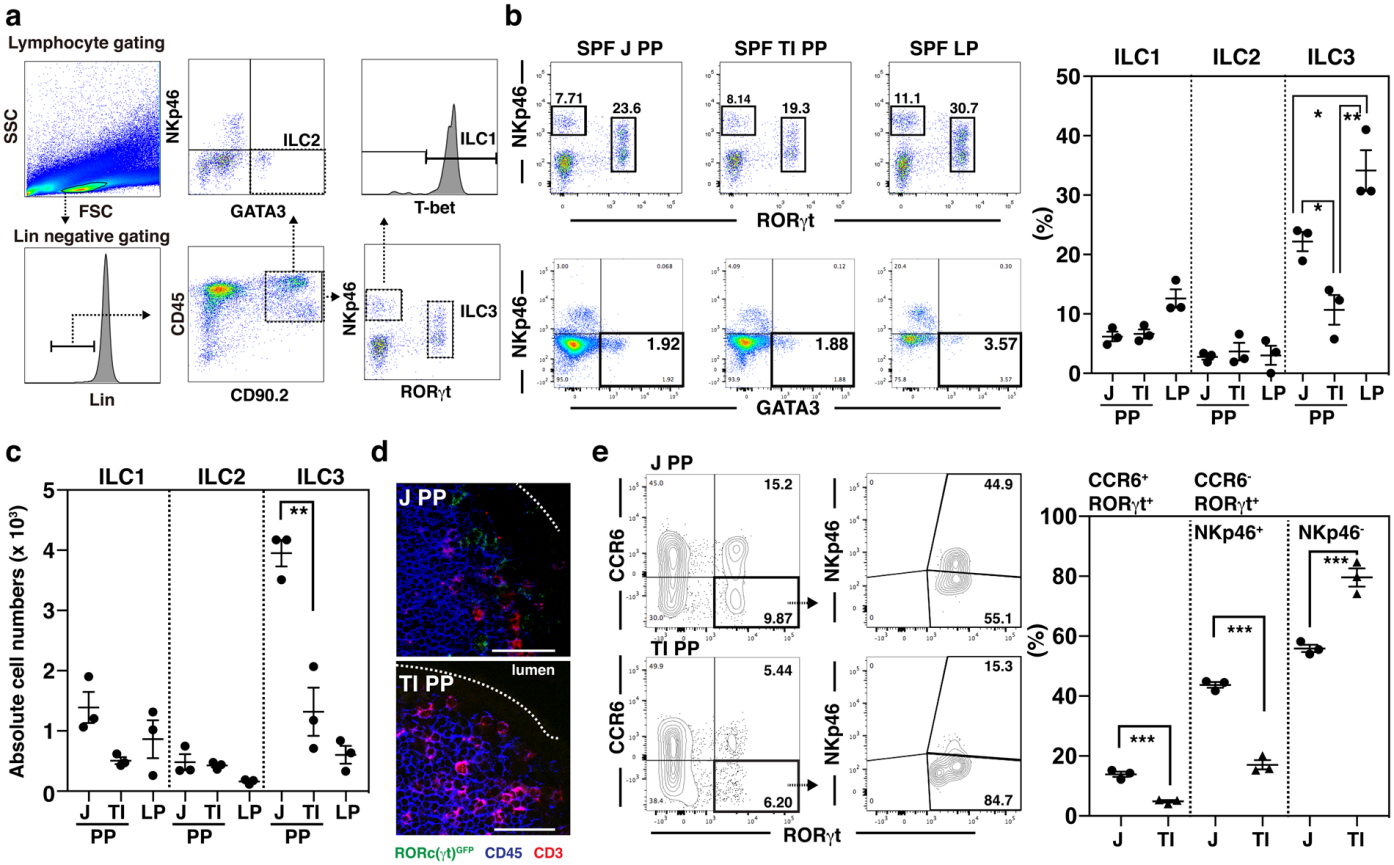
Terminal ileal PP has fewer ILC3s than jejunal PP. (**a**) ILCs were categorized into ILC1s, ILC2s, and ILC3s using a gating strategy such that lin^−^RORγt^−^ NKp46^+^ T-bet^+^, lin^−^ NKp46^−^ GATA3^+^, and lin^−^ RORγt^+^ NKp46^+/−^ PP cells represented ILC1s, ILC2s, and ILC3s, respectively. (**b**) The distribution of ILCs in PPs of the terminal ileum (TI PP), jejunum (J PP), and lamina propria (LP) of SPF mice was analyzed by flow cytometry according to the gating strategy above. The numbers indicate the percentage of cells in each gated area and data are representative of three independent experiments. (**c**) The absolute numbers of ILC1s, ILC2s, and ILC3s were counted from PP cells of the terminal ileum (TI PP), jejunum (J PP), and lamina propria (LP) in SPF mice, as explained in the Methods. (**d**) The CD3^−^ CD45^+^ (blue) RORγt^*gfp/*+^ (green) ILC3 cells were analyzed in cryosectioned slices of jejunal and terminal ileal PPs, prepared from RORc(γt)^gfp^ reporter mice, by confocal laser scanning microscopy. The scale bar represents 50 μm and the dotted line depicts the FAE of PPs. (**e**) The frequencies of ILC3 subtypes (CCR6^+^, CCR6^−^ NKp46^+^, and CCR6^−^ NKp46^−^) were analyzed from jejunal and terminal ileal PP cells of SPF mice by flow cytometry. Data represent the mean ± SE calculated from three independent experiments with three mice per group. *p < 0.05, **p < 0.01, ***p < 0.001 indicate significant differences between the groups compared.

The populations and frequencies of immune cells in the intestinal mucosal compartment are regionally specialized^18^. For example, Th17 cell numbers decrease gradually from the jejunum to the ileum in the small intestine and FoxP3^+^ Tregs increase gradually from the small intestine to the colon^30^. Although the number of ILC3s increases from the proximal to distal LPs in the small intestine, little information is available regarding the distribution of ILCs depending on anatomical PP location^25^. Because PPs scattered within the small intestine are exposed to various environments, in terms of the species and the numbers of microbiota, we considered that PP cells would have distinctive distributions. To this end, we analyzed the regionally specialized distribution of ILCs in PPs of the jejunum and terminal ileum according to an ILC gating strategy based on specific transcription factor expression (Fig. 1a–c).

In specific pathogen-free (SPF) mice, the absolute number of lin^−^CD45^+^CD90.2^+^ RORγt^+^ cells (ILC3s) in the terminal ileal PP was 943.9 ± 235, which was at least 4-fold lower than that in the jejunal PP (3,841 ± 331); this difference was not detected in other groups of ILCs (Fig. 1c). Using RORc(γt)^*gfp*^ reporter mice, we confirmed the distribution of CD3^−^RORγt^*gfp/*+^ ILC3s to be localized in the subepithelial dome of jejunal PPs, but not in terminal ileal PPs (Fig. 1d). We further analyzed the PP ILC3s based on the expression of cell surface markers, CCR6 and NKp46 (Fig. 1e). The frequency of LTi-like CCR6-expressing cells and CCR6^−^NKp46^+^ cells in the terminal ileal PPs was at least 2-fold lower (*p* < 0.001) than that in jejunal PPs. In contrast, the frequency of CCR6^−^NKp46^−^ cells in terminal ileal PPs was significantly higher (*p* < 0.001) than that in jejunal PPs. These results indicate that the frequency of ILC3s in PPs is influenced by the regional environment of the PPs. Importantly, given that a failure of ILC3 homeostasis results in various disease states, including inflammatory diseases and cancers^31, 32^, it is reasonable to think that identification of the regionally specialized factor(s) in terminal ileal PPs capable of modulating ILC3s may help in determining the mechanism of homeostatic maintenance in mucosal immunity.

### The microbiota is a regionally specialized factor modulating ILC3s in terminal ileal PPs

**Figure 2:**
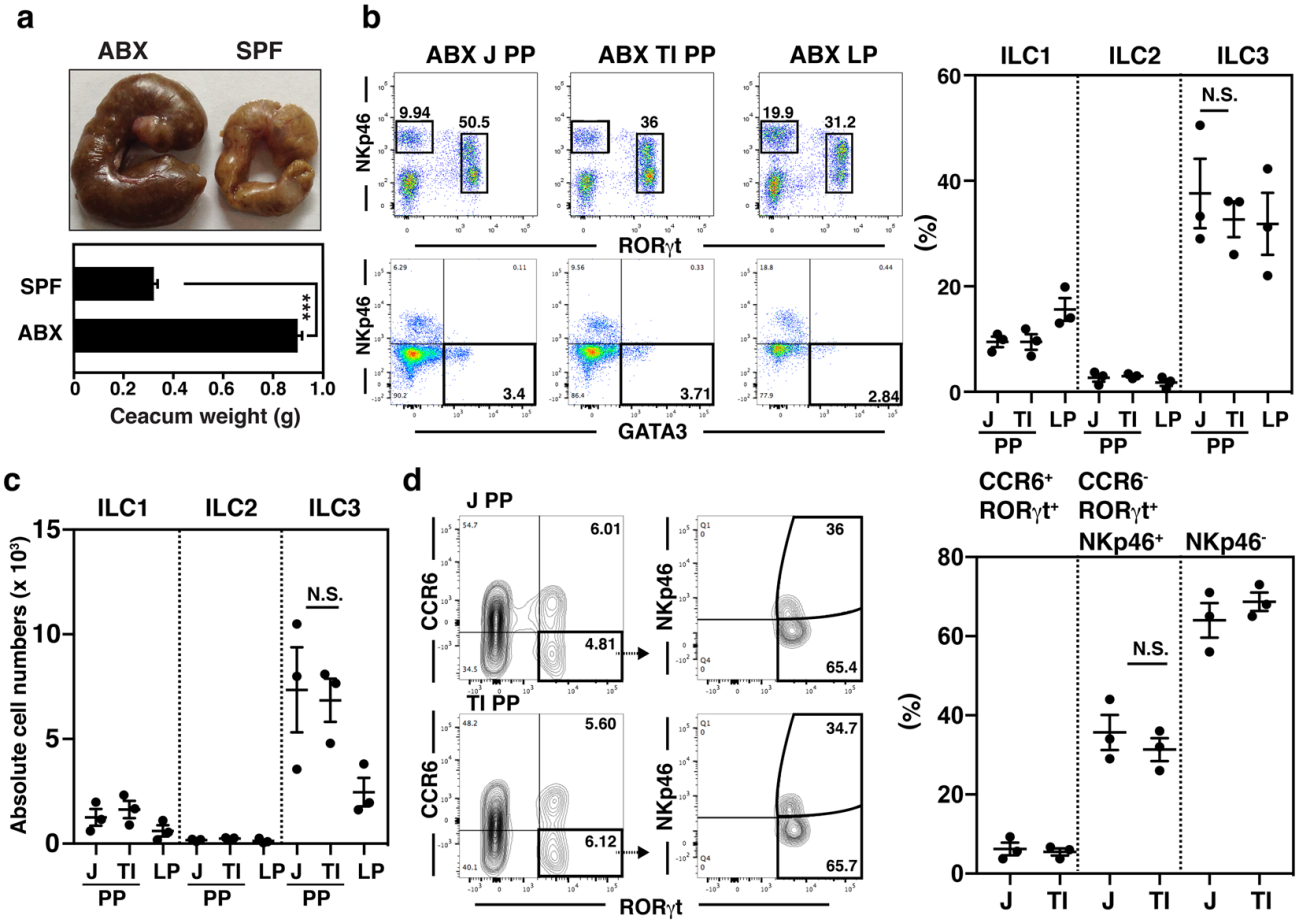
The microbiota exerts an indirect influence on the negative regulation of ILC3s in terminal ileal PPs. (**a**) The size and weight of ceca from SPF and ABX-treated mice were compared. (**b**) The distribution of ILCs in PPs of terminal ileum (TI PP), jejunum (J PP), and lamina propria (LP) of ABX-treated mice was analyzed by flow cytometry, according to the gating strategy explained in Fig. 1. The numbers indicate the percentage of cells in each gated area and data representative of three independent experiments are shown. (**c**) The absolute numbers of ILC1s, ILC2s, and ILC3s were counted from PPs of terminal ileum (TI PP), jejunum (J PP), and lamina propria (LP) in SPF mice, as explained in the Methods. (**d**) The frequencies of ILC3 subtypes (CCR6^+^, CCR6^−^ NKp46^+^, and CCR6^−^ NKp46^−^) were analyzed in jejunal and terminal ileal PP cells of ABX-treated SPF mice by flow cytometry. Data represent the mean ± SE calculated from three independent experiments with three mice per group.

To identify the major factor(s) limiting ILC3 distribution in terminal ileal PPs, we first considered the influence of T cells, based on a previous suggestion of a negative effect of T cells on ILC expansion^33^. However, the influence of T cells can be excluded using BALB/c nude mice because the distinctive pattern of ILC3 distribution in PPs of the terminal ileum and jejunum was observed similarly in nude mice (Supplementary Fig. 1). We next investigated the role of the microbiota as a major factor limiting ILC3 distribution in terminal ileal PPs, based on a previous study showing that microbiota repressed the production of IL-22 derived from ILC3s, although it was controversial whether the microbiota was influential in ILC3 development^26, 33^. To eliminate the influence of microbiota, mice were treated with antibiotics (ABX) for 4 wk and these ABX-treated mice showed enlarged ceca compared with those of SPF mice^12^, which is a reflection of the decrease in microbiota by ABX treatment (Fig. 2a). Importantly, the absolute number of ILC3s in the terminal ileal PPs from ABX-treated mice was 6,015 ± 2,000, which was similar to that in the jejunal PPs (6,588 ± 2,300), but the distinctive difference in frequency in the distribution of ILC3s between jejunal and terminal ileal PPs remained in SPF mice (Fig. 2b,c and Supplementary Fig. 2a). The frequency of CCR6^+^ and CCR6^−^NKp46^+^ ILC3s was also increased among ILC3s of terminal ileal PPs, but the significantly distinctive difference in the frequency of NKp46^+^ and NKp46^−^ ILC3s remained in SPF mice (Fig. 2d and Supplementary Fig. 2b). These results suggest that all subsets of ILC3s in PPs are regulated by the microbiota, although it was unclear whether the influence exerted by the microbiota was direct or indirect.

### Microbiota-derived butyrate is a factor directly limiting the number of ILC3s in terminal ileal PPs

**Figure 3:**
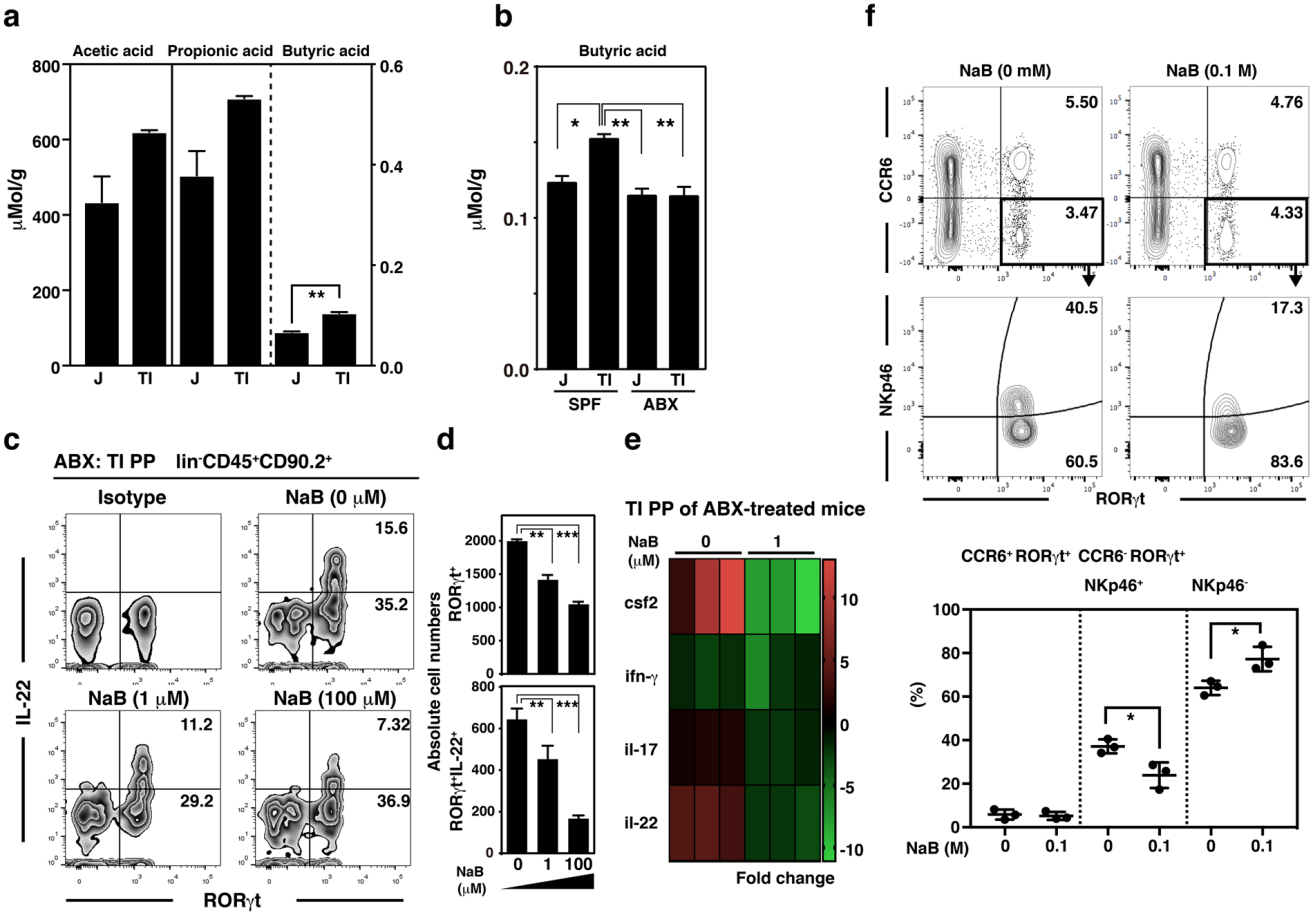
Butyrate exerted a suppressive effect on ILC3s in PPs of the terminal ileum. (**a**) The levels of SCFAs in jejunum (J) and terminal ileum (TI) were determined by GC-MS. (**b**) The levels of butyric acid were measured in jejunal PP and terminal ileal PP from SPF mice and ABX-treatment SPF mice. (**c**) The level of IL-22 in lin^−^ CD45^+^CD90.2^+^ cells prepared from terminal ileal PPs of ABX-treated mice that had been stimulated *in vitro* with butyrate (NaB) for 17 hr and re-stimulated with PMA, ionomycin, and IL-23 was determined as described in the Methods. The numbers indicate the percentage of cells in each gated area and data representative of three independent experiments are shown. (**d**) Absolute numbers of RORγt-expressing cells (upper panel) and cells expressing both RORγt and IL-22 (lower panel) were counted using the experimental procedure described above. (**e**) The RORγt^*gfp/*+^ cells were sorted from terminal ileal PPs prepared from ABX-treated RORc(γt)^gfp^ reporter mice. Levels of transcripts for the cytokines indicated were measured in sorted RORγt^*gfp/*+^ cells treated with or without NaB (1 μM) using a PCR array. The fold change values were calculated by comparing the values with those obtained from terminal ileal PP ILCs prepared from SPF C57BL/6 mice or RORγt^*gfp/*+^ or sorted RORγt^gfp^ cells without NaB treatment. (**f**) Changes in the frequency of ILC3 subpopulations in jejunal and terminal ileal PPs of ABX-treated mice were determined with or without the oral administration of NaB (0.1 M). The numbers indicate the percentage of cells in each gated area and data representative of three independent experiments are shown. Data represent the mean ± SE counted from three independent experiments with three mice per group. *p < 0.05, **p < 0.01, and ***p < 0.001 indicate significant differences between the groups compared.

Although an interaction between ILC3s and the intestinal microbiota was already identified, it was unclear how ILC3s received signals derived from the microbiota. Given that mouse ILC3s do not express Toll-like receptors, products derived from bacterial metabolism, including SCFAs, are possible regulators of ILC3s^34^. Because the enlarged ceca seen in ABX-treated mice were due to the accumulation of hydrated dietary fiber components, we assessed whether the level of SCFAs showed a distinctive pattern between jejunal PPs and terminal ileal PPs. When we measured the level of SCFAs using gas chromatography-mass spectrometry (GC-MS), the levels of SCFAs in terminal ileal PPs were higher than those in jejunal PPs (Fig. 3a). In particular, the level of butyric acid was significantly higher in terminal ileal PPs than in jejunal PPs. Given that butyrate is a major metabolic product of colonic bacteria, some of which also colonize the terminal ileum, these results made sense^12^. We investigated the biological function of SCFAs on ILC3s by treating jejunal PP cells with each metabolite and found that only sodium butyrate treatment induced a significant decrease (*p* < 0.05) in IL-22 expression in RORγt^+^ ILC3s (Supplementary Fig. 3). Consequently, we believed that butyrate was a regionally specialized factor, suppressing ILC3s in terminal ileal PPs. Indeed, the level of butyrate in terminal ileal PPs of SPF mice was significantly higher (*p* < 0.05) than that in jejunal PPs and ABX treatment significantly reduced (*p* < 0.01) the level of butyrate in terminal ileal PPs, similar to that in jejunal PPs (Fig. 3b).

We next assessed whether butyrate directly inhibited the expression of RORγt and/or IL-22 in ileal PP cells from ABX-treated mice (Fig. 3c). Butyrate treatment not only decreased the number of RORγt^+^ PP cells, at least 2-fold, but also reduced IL-22 expression significantly in a dose-dependent manner (Fig. 3c,d). To confirm the direct effect of butyrate on ILC3s, the level of cytokine transcripts was monitored after *in vitro* butyrate treatment of sorted RORγt^*gfp/*+^ ILC3s prepared from terminal ileal PPs of ABX-treated RORc(γt)^*gfp/*+^ mice (Fig. 3e). RORγt is a master transcription factor for Th17-type cytokines and sorted RORγt^*gfp/*+^ ILC3s expressed high levels of *Csf2*, *Il-17*, and *Il-22* transcripts compared with RORγt^−^ ILCs. Importantly, the high-level expression of the transcripts was decreased at least 5-fold by butyrate treatment. We next analyzed whether restoring the butyrate level in ABX-treated mice induced changes in the ILC3 distribution in terminal ileal PPs and found that the frequency of ILC3s was decreased significantly in jejunal and terminal ileal PPs by the oral administration of butyrate (Supplementary Fig. 4). Additionally, the frequency of RORγt^+^CCR6^−^NKp46^+^ cells was also decreased significantly (*p* < 0.05) by butyrate treatment, as was seen in SPF mice, although the frequency of RORγt^+^CCR6^−^NKp46^−^ was increased by butyrate treatment (Supplementary Fig. 4 and Fig. 3f). Taken together, these results suggest that butyrate acts as a regionally specialized factor specifically on ILC3s, and that NKp46^+^ ILC3s in terminal ileal PPs are repressed by microbiota-derived butyrate under steady-state conditions.

### Butyrate modulates the plasticity of NKp46^+^ ILC3s via GPR109a-mediated signaling

**Figure 4:**
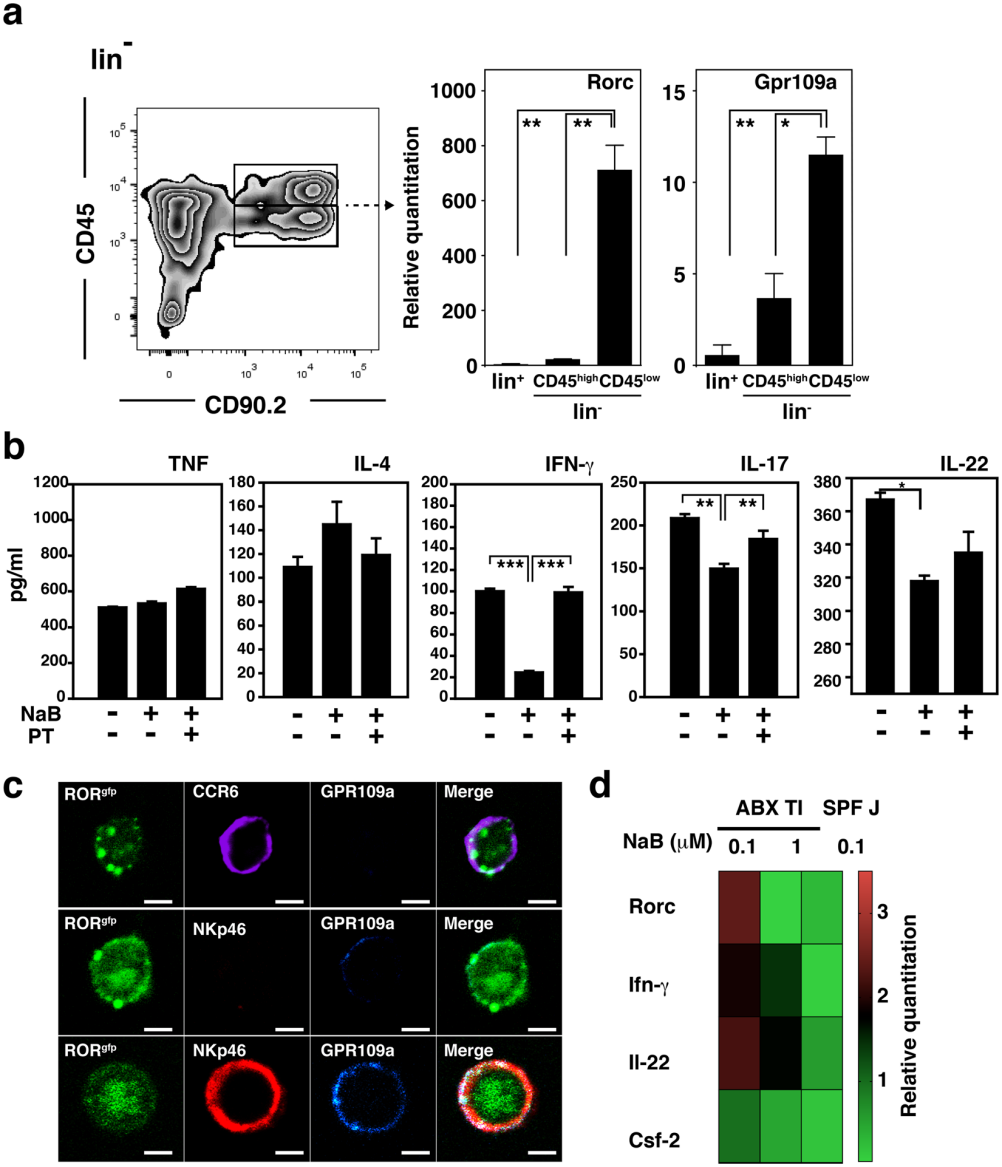
NKp46^+^ ILC3s responded to butyrate stimulation through GPR109a-mediated signaling. (**a**) Expression levels of mRNAs for *Rorc* and *Gpr109a* were determined from lin^+^, lin^−^CD45^high^ CD90.2^+^, and lin^−^CD45^low^CD90.2 cells sorted from PP cells from SPF mice. Data represent the mean ± standard deviation of three mice per group and a representative result from three independent experiments is shown. *p < 0.05 and **p < 0.01 indicate significant differences between the groups compared. (**b**) Levels of cytokines secreted from ILCs prepared from PPs of BALB/c nude mice stimulated with the molecules indicated and re-stimulated with PMA, ionomycin, and IL-23 were determined as described in the Methods. (**c**) The sorted lin^−^RORγt^*gfp/*+^ cells were stained with anti-CCR6 Ab, anti-NKp46 Ab, or anti-GPR109a Ab and assessed using confocal laser scanning microscopy. The scale bars represent 5 μm. (**d**) The lin^−^RORγt^*gfp/*+^ cells were prepared from terminal ileal PPs of ABX-treated SPF mice or jejunal PPs of SPF mice and stimulated *in vitro* with each indicated concentration of NaB for 2 hr. Levels of the listed cytokine transcripts were then measured using a quantitative real-time PCR assay. Data from three independent experiments are presented as a heat map and relative quantitation (RQ) of cDNA was calculated by the ΔΔCt method using 18S rRNA genes. Data represent the mean ± SE measured from three independent experiments. *p < 0.05, **p < 0.01, and ***p < 0.001 indicate significant differences between the groups compared.

We next tried to characterize the mechanism of the butyrate-mediated regulation of ILC3s by analyzing intracellular signals involved in this repression. GPR109a, a receptor for butyrate, was expressed highly in RORγt^+^ PP ILC3s, which are lin^−^CD45^low^CD90.2^+^ PP cells, and were additionally validated by the expression level of the *Rorc* transcript^13^ (Fig. 4a). To assess whether butyrate could inhibit the activity of ILC3s via GPR109a-mediated Gαi signaling, lin^−^CD45^+^CD90.2^+^ PP cells were pretreated with the Gαi inhibitor pertussis toxin (PT), followed by stimulation with butyrate. Cytokine levels were then measured after co-treatment with phorbol 12-myristate 13-acetate (PMA) and ionomycin in the presence or absence of IL-23 (Fig. 4b). The levels of ILC3-derived cytokines, such as IFN-γ, IL-17, and IL-22, were decreased by butyrate treatment and these decreases were abrogated by PT pretreatment, although the degree of abrogation was statistically insignificant in IL-22. In contrast, the same treatment did not affect the expression level of cytokines, such as TNF and IL-4, which are expressed by another group of ILCs (Fig. 4b). Based on these results, we considered that ILC3s in PPs expressed functional GPR109a and that butyrate induced plasticity of ILC3s via GPR109a signaling. We further confirmed the expression of GPR109a in RORγt^*gfp/*+^ cells expressing CCR6 or NKp46 (Fig. 4c). Consistent with the results from flow cytometric analysis of the cells in jejunal and ileal PPs, we found that RORγt^*gfp/*+^NKp46^+^ ILC3s expressed higher levels of GPR109a than RORγt^gfp/+^CCR6^+^ ILC3s or RORγt^gfp/+^NKp46^−^ ILC3s. To further confirm the GPR109a-mediated signaling mediated by butyrate, RORγt^gfp/+^NKp46^+^ ILC3s were sorted from terminal ileal PPs of ABX-treated mice or jejunal PPs of SPF mice. Butyrate treatment caused a decrease in *Rorc*, *Ifn-γ*, *Il-22*, and *Csf2* transcripts from sorted RORγt^gfp/+^NKp46^+^ ILC3s (Fig. 4d). Collectively, these results suggest that butyrate regulates the plasticity of NKp46^+^RORγt^+^ ILC3s through inhibition of *Rorc* expression via GPR109a signaling.

### Negative regulation by butyrate can contribute to the construction of an ILC3-Treg axis to maintain homeostasis in terminal ileal PPs

**Figure 5:**
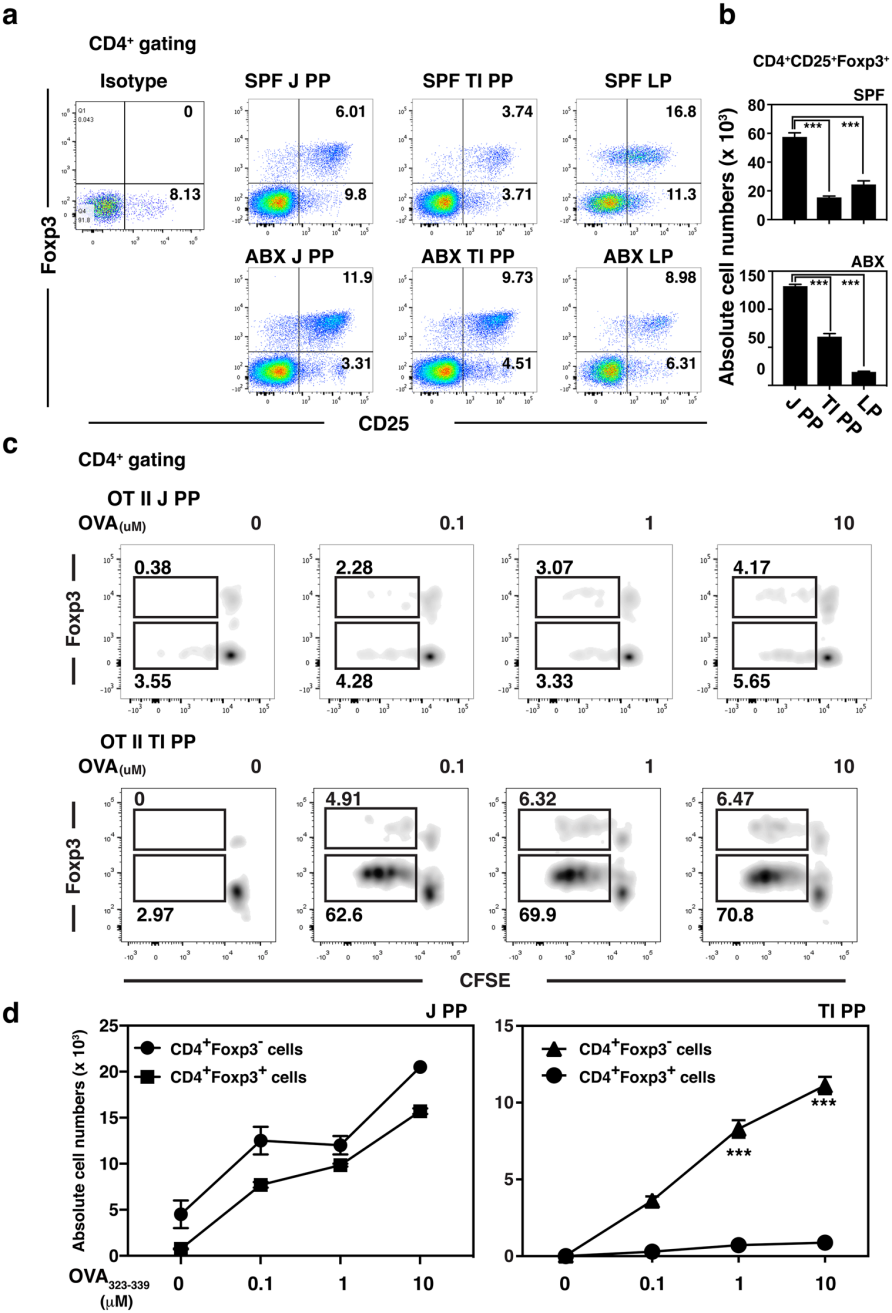
Suppression of ILC3s by butyrate treatment negatively affected Tregs in PPs. (**a**) Distributions of CD4^+^CD25^+^Foxp3^+^ Tregs in PPs of the terminal ileum (TI PP), PPs in the jejunum (J PP), and the LP of SPF mice (upper panel) and ABX-treated mice (lower panel) were analyzed by flow cytometry. The numbers indicate the percentage of cells in each area and data representative of three independent experiments are shown. (**b**) Absolute numbers of CD4^+^CD25^+^Foxp3^+^ Tregs in PPs of the terminal ileum (TI PP), PPs in the jejunum (J PP), and LP of SPF mice (upper panel) and ABX-treated mice (lower panel) are shown. Data represent the mean ± SE counted from three independent experiments with two mice per group. (**c**) *In vitro* proliferation of CD4^+^Foxp3^+^ cells in PPs of the terminal ileum (TI PP) and PPs of the jejunum (J PP) from transgenic OT-II mice by OVA peptide treatment was analyzed by flow cytometry. The numbers indicate the percentage of cells in each area and data representative of two independent experiments are shown. (**d**) The absolute numbers of CD4^+^Foxp3^+^ Tregs and CD4^+^Foxp3^−^ T cells were counted from experiment (**c**). Data represent the mean ± SE counted from three independent experiments with two mice per group.

Recent studies have shown a close relationship between ILC3s and tolerance to microbiota, such that ILC3s express CSF2 by IL-1β, secreted by macrophages stimulated with microbiota, and CSF2-primed DCs promote local Treg homeostasis^28^. Consequently, we sought to determine the influence of reduced ILC3 frequency in terminal ileal PPs on mucosal adaptive immune response induction. Based on the observation that one of the cytokines with prominently reduced expression in terminal ileal PP cells was ILC3-derived CSF2, which is essential for Treg maintenance in the steady-state colon (Fig. 3e), we considered that the low ILC3 number may reduce the Treg frequency in terminal ileal PPs. When we measured the distribution of CD4^+^CD25^+^Foxp3^+^ Tregs in jejunal and terminal ileal PPs, and the LP from SPF mice, we found at least 10-fold decreased numbers of Tregs in terminal ileal PPs compared with those in jejunal PPs of SPF mice (Fig. 5a,b). This difference in the number of Tregs in PPs of terminal ileum and jejunum was reduced by ABX treatment, although the difference was still significant. These observations prompted us to consider that low numbers of ILC3s and Tregs may contribute to the induction of Ag-specific immunity in the steady-state SPF mice. When we prepared PP cells from terminal ileum and jejunum of transgenic OT-II mice and monitored the proliferation of CD4^+^Foxp3^−^ or CD4^+^Foxp3^+^ T cells induced by OVA peptide treatment after carboxyfluorescein succinimidyl ester staining, OVA-specific CD4^+^Foxp3^−^ T cells in terminal ileal PPs, where low numbers of ILC3s and Tregs were present, proliferated significantly more (*p* < 0.001) than OVA-specific CD4^+^Foxp3^+^ T cells, in a dose-dependent manner (Fig. 5c,d). In contrast, the significant difference in the proliferation of OVA-specific CD4^+^Foxp3^−^ and OVA-specific CD4^+^Foxp3^+^ T cells was not detected in jejunal PPs and both cell types proliferated effectively with OVA peptide stimulation. These results suggested that the terminal ileum establishes a unique microenvironment capable of regulating Ag-specific immune response induction through maintaining low numbers of ILC3s and CD4^+^Foxp3^+^ Tregs.

## Discussion

In the gastrointestinal tract, the regional diversity of intestinal contents, including dietary components, microbes, and microbial metabolites, affects the frequency and distribution of mucosal immune cells^18^. However, the regional specialization of the cells in PPs, a key mucosal immune organ where regulation of the linkage and balance between innate and adaptive immunity occurs, along the gastrointestinal tract, is not clearly understood. It could be speculated that PPs in the jejunum, where the digestion and absorption of food typically take place, need to maintain a tolerogenic microenvironment toward food Ags, while PPs in the terminal ileum, which are exposed to massive amounts of microbes compared with those in the jejunum, need to create an immunogenic microenvironment for the induction of Ag-specific immune responses^18, 35^. We considered that ILCs function as regulatory cells in this complex cellular network and confirmed, in this study, that butyrate, the regional specialized factor of PPs, does affect ILC distribution. Additionally, the distinctive ILC distribution is closely associated with the induction of Ag-specific immune responses.

Although it is still controversial, the microbiota has an influence on the frequency of ILC3s in the gut^26^. For example, germ-free mice have been shown to exhibit a decrease in IL-22-expressing NKp46^+^ ILC3s^36^, whereas other studies have shown that the microbiota plays a role as a negative regulator of ILC3s in the LP of the small intestine^33^. We also showed a role of the microbiota as a negative regulator on PP ILC3s, especially on NKp46^+^ ILC3s. Although the influence of the microbiota on ILC3s has been recognized, little is known about how ILC3s sense signals from the microbiota. Given that ILC3s do not express Toll-like receptors, by-products of microbial metabolism are candidate molecules of ILC regulation^26^. For example, among various metabolites produced by microorganisms, retinoic acid, which is concentrated primarily in the small intestine, drives the maintenance of RORγt^+^ ILC3s, while it also suppresses the expansion of ILC2s^37^. In contrast, SCFAs can enhance the numbers and function of FoxP3^+^ Tregs in the colon^12^. Our results showed that butyrate treatment, *in vitro* and *in vivo*, contributed to the decrease in RORγt^+^ ILC3s in PP cells (Fig. 3). This negative effect could be achieved by various mechanisms, such as induction of apoptosis, inhibition of cell proliferation, and regulation of master transcription factor(s). We found that the level of annexin V was unchanged on the surface of ILC3s after butyrate treatment (data not shown). Although the ILC3 proliferation can be supported by IL-1β, a quick decrease in *Rorc* transcripts in NKp46^+^RORγt^*gfp/*+^ cells, at 2 hr after *in vitro* butyrate treatment, may suggest that the suppressive effect of butyrate on ILC3s is closely related to the regulation of *Rorc* transcript expression (Fig. 4d). RORγt expression in ILC3s is unstable and is affected by the local environment, such as the microbiota and cytokines^38^. For example, loss of RORγt expression in NKp46^+^ ILC3s was induced by T-bet upregulation, which, in turn, generated ex-ILC3s, while RORγt expression was stabilized by IL-7 signaling^25, 39^. In NKp46^+^ ILC3s, TGF-β signaling induced the loss of NKp46, which, in turn, generated NKp46^−^ ILC3s^40^. Additionally, IL-23 signaling drives NKp46 expression in NKp46^−^ ILCs, which, in turn, revert to NKp46^+^ ILC3s^41, 42^. In this study, we showed that ABX treatment made the microenvironment of the terminal ileal PPs similar to that of jejunal PPs, through decreasing the butyrate level and increasing the number of ILC3s (Fig. 2). Notably, this change was seen in mice treated with ABX for at least 4 wk. Thus, we suggest that the change in the number of ILC3s in the terminal ileal PPs induced by ABX treatment, which removes the source of butyrate in the small intestine, resulted from the maintenance of ILC3s due to the absence of a butyrate-mediated inhibitory signal for RORγt expression. Collectively, our findings on the relationship between butyrate and PP ILC3 regulation may also represent the ILC3 plasticity and indicate a new feature of the regulation of mature ILC3s.

It was notable that the fermentation end products of *Clostridium* spp., which are found mainly in the terminal ileum and colon, are acetate and butyrate and that *Alcaligenes* spp. reside in ileal PPs, where they produce butyrate^43, 44^. Differences in spatially localized microbe species and/or their metabolites clearly imply imprinting effects within ILCs of the gastrointestinal tract by regional specialization. Here, we suggest butyrate as a regionally specialized factor of terminal ileal PPs that regulates ILC3s. The butyrate concentration in the colonic lumen is 10–20 mM and is sufficient as an endogenous agonist for GPR109a signaling^45^. We showed that terminal ileal PPs contain a higher level of butyrate than jejunal PPs (Fig. 2). Although the overall butyrate level in terminal ileal PPs is much lower than that in the colonic lumen and that required to activate the signaling through GPR109a, we showed that 10 μM butyrate can activate GPR109a in NKp46^+^ ILC3s (Fig. 4). Consequently, it seems reasonable that NKp46^+^ ILC3s are sensitive to butyrate signaling and the difference in butyrate concentrations between terminal ileal PPs and jejunal PPs could be a factor leading to the distinguishable biological response induction. The niacin/butyrate receptor GPR109a is expressed in intestinal epithelial cells, DCs, and macrophages in the colon, but its expression had not been investigated previously in NKp46^+^ ILC3s^46^. We confirmed the functional expression of GPR109a on RORγt^+^ ILC3s in PPs and showed that a decrease in ILC3-derived cytokines occurred after butyrate treatment (Fig. 4b). In fact, it is important to note that failure of ILC3 homeostasis results in various disease states, including chronic inflammatory diseases and some cancers^47^. For example, IFN-γ-producing group 1 ILCs (ILC1s) and ILC3s increased disease severity in a colitis model, and IL-17-producing ILC3s have been shown to be closely associated with inflammatory bowel disease^48, 49^. It has also been reported that ILC3-derived IL-22 promotes the proliferation of colorectal cancer cells^50^. However, because the function of negative regulators of RORγt^+^ ILC3s, such as IL-25, is poorly understood, our observation of butyrate-mediated negative regulation of PP ILC3s is an important finding with respect to the regulation of the mucosal immune system.

Interaction between ILC3s and the intestinal microbiota also affects the functions of other mucosal cells^51^. For example, IL-22-producing ILC3s induce fucosylation on epithelial cells in the ileum; RORγt-deficient mice showed disrupted intestinal fucosylation and increased infection by *Salmonella typhimurium* ^52^. ILC3s prevent the dissemination of ileal PP-resident *Alcaligenes* spp. into the systemic immune compartment and systemic inflammation^29^. Additionally, LTα1β2-expressing ILC3s localized in the subepithelial dome of PPs play a key role in controlling B-cell isotype class switching to IgA-producing cells through CSF2 expression by contacting CD11c^+^ DCs^27^. In fact, CSF2 expressed from ILC3s is closely related to the regulation of mucosal adaptive immunity through Treg maintenance^53^. For example, CSF2-deficient mice showed a reduced number of Tregs in the LP and impaired oral tolerance^28^. Based on this, we suggest that the decrease in CSF2, caused by the reduction in ILC3s in terminal ileal PPs, may be related to the observed difference in the ratio of Tregs in jejunal and terminal ileal PPs. In this study, ABX treatment increased the number and/or frequency of Tregs in PPs. In that sense, regional specialization of ILC3s differentially affects adaptive immunity in jejunal PP and in terminal ileal PPs. The induction of Ag-specific immune responses in the gut LP is sensitively regulated against numerous Ags continuously entering into the lumen and the microbiota to maintain a tolerogenic condition, so as not to cause an excessive inflammatory response^54, 55^. This predominant negative regulation of the induction of Ag-specific immune responses in gut LP is achieved through Treg proliferation by Ag-presenting cells, including CX3CR1-expressing macrophage cells and CD103^+^ DCs^56^. In comparison with the LP, the PP, a key organ in the induction of Ag-specific IgA responses, requires tight regulation of the balance between tolerogenic and immunogenic responses^57^. In that sense, it is conceivable that using a small cell population capable of modulating Tregs, like ILCs, would be a useful strategy to regulate the microenvironment of PPs. The suppression of the proliferation of Tregs in the terminal ileal PPs, but not in jejunal PPs, by Ag stimulation supports a regulatory role for butyrate in the ILC3-Treg axis of the immune response (Fig. 5d). In fact, it is known that butyrate (100 μM) treatment of naïve T cells for 3 d drives Treg differentiation through chromatin condensation^12^. We also agree that butyrate can induce the differentiation and activation of Tregs in PPs and suggest that ILC3-derived CSF2 contributes to maintaining the number of Tregs in PPs, which does not imply that butyrate suppresses Tregs.

Taken together, our results suggest the regionally specialized localization of ILC3s in the PPs of the terminal ileum and demonstrate that the number of ILC3s is suppressed by microbe-derived butyrate via GPR109a in PPs of SPF mice under steady-state conditions. This suppression of ILC3s in terminal ileal PPs, in comparison with the jejunum, results in decreasing the level of CSF2, which, in turn, limits the number of Tregs. Finally, this biological consequence of ILC3-Treg axis immune regulation in PPs may be related to Ag-specific immune response induction.

## Methods

### Experimental materials and mice

All chemicals were purchased from Sigma Chemical Co. (St. Louis, MO), unless indicated otherwise. Syngeneic BALB/c, BALB/c nude, and C57BL/6 mice were purchased from Charles River Technology through Orient Bio (Sungnam, Korea). Transgenic OT-II mice and RORc(γt)^gfp^ reporter mice were kindly provided by Dr. M. Song (International Vaccine Institute, Seoul, Korea) and Dr. Charles D. Surh (Pohang University of Science and Technology, Pohang, Korea), respectively. To prepare ABX-treated mice, mice were given a cocktail of ABX (1 g/L ampicillin, 0.5 g/L vancomycin, and 0.1 g/L polymyxin), purchased from Duchefa Biochemie (Haarlem, The Netherlands), in drinking water for 4 wk.

Experimental procedures involving laboratory animals were approved by the Institutional Animal Care and Use Committee of the Chonbuk National University (Approval Numbers: CBU 2011-0061 and CBU 2015-0004). They were carried out in accordance with the guidelines of the committee.

### Flow cytometry

PP and LP cells were isolated from the small intestine as described previously^58^. All antibodies (Abs) used for flow cytometric analyses were purchased from BD Biosciences, unless indicated otherwise. Prepared cells were pre-treated with Mouse BD Fc Block (BD Pharmingen, Franklin Lakes, NJ). For the analysis of transcription factors, cells were initially fixed and permeabilized with a transcription factor buffer set (BD Pharmingen). The cells were then stained with PerCP-Cy5.5 mouse lineage Ab cocktail (CD3e, Cd11b, CD45R, Ly-76, Ly-6G, Ly-6C), PE-conjugated anti-T-bet Ab, PE-CF594-conjugated anti-RORγt Ab, or PE-CF594-conjugated anti-GATA-3 Ab, followed by FITC-conjugated anti-CD335 Ab, allophycocyanin (APC)-conjugated anti-CD90.2 Ab, and PE-Cy7-conjugated anti-CD45 Ab. For intracellular cytokine staining of Foxp3, cells were fixed, permeabilized, and stained with FITC-conjugated anti-CD4 Ab, PE-conjugated anti-CD25 Ab, and APC-conjugated anti-Foxp3 Ab using a Foxp3 staining set (eBioscience, San Diego, CA), according to the manufacturer’s protocol. For intracellular cytokine staining of IL-22, PP cells were cultured with or without butyrate and then stimulated for 4 hr with PMA (50 ng/mL), ionomycin (500 ng/mL), and mouse recombinant IL-23 (40 ng/mL; R&D Systems, Minneapolis, MN). Brefeldin A (eBioscience) was added to the culture medium and cells were incubated for the final 1.5 hr. Cells were then fixed, permeabilized, and stained with PerCP-Cy5.5 mouse lineage Ab cocktail, PE-CF594-conjugated anti-RORγt Ab, and APC-conjugated anti-IL-22 Ab (eBioscience), followed by APC-eFluor780-conjugated CD90.2 Ab and PE-Cy7-conjugated anti-CD45 Ab using a transcription factor buffer set, according to the manufacturer’s protocol. The absolute cell number was calculated using 123-count eBeads (eBioscience), according to the manufacturer’s protocol.

To measure the proliferation of Foxp3^+^ cells, whole PP cells were stained with CSFE (eBioscience) according to the manufacturer’s protocol and the stained cells were cultured for 4 d with OVA_323–339_ peptide. Similarly, PP cells from wild-type C57BL/6 mice were stimulated with OVA_323–339_ peptide, inactivated by mitomycin C treatment, and then co-cultured with CFSE-stained CD4^+^ T cells prepared from splenocytes of OT-II mice. After stimulation, cells were stained with PerCP-Cy5.5-conjugated anti-CD4 Ab (BD Biosciences) and APC-conjugated anti-Foxp3 Ab (eBioscience), and analyzed by flow cytometry.

### Gas chromatography-mass spectrometry (GC-MS)

The organic acid concentrations in the terminal ileum, jejunum, and PPs were determined using GC-MS. Briefly, tissues were homogenized in extraction solution containing 2 N HCl. After vortexing, homogenates were centrifuged (13,000 rpm, 10 min). The supernatant was transferred to a new glass vial, followed by the addition of NaCl until saturation was reached, and then ethyl acetate was added and the mixture was centrifuged. The top layer was collected, transferred to a new glass vial, and dried using N_2_. The dried samples were mixed with N-tert-butyldimethylsilyl-N-methyltrifluoroacetamide and analyzed. Organic acid concentrations were quantified by comparing their peak areas with standards of known concentrations.

### Quantitative real-time PCR

Total RNA was purified using the RNeasy Plus micro kit (Qiagen, Valencia, CA) according to the manufacturer’s protocol and cDNA was prepared using the RT^2^ first strand kit (Qiagen). For the analysis of receptor and transcription factor gene expression in ILCs, the following primers were purchased from Qiagen: *Gapdh* (PPM02946E), *Gpr43* (PPM04863A), *Gpr41* (PPM59038A), and *Rorc* (PPM25095A). The *Gpr109a* (NM_030701) gene was amplified using the following primer set: forward primer, 5′-ACC CTA GGA CGA AGA GCC AT-3′ and reverse primer, 5′-TTT GAC TCC CAG ATG CAC CC-3′. cDNA was analyzed using an ABI 7500 system (Life Technology, Carlsbad, CA) and the expression level of each gene was normalized to the level of Gapdh expression using a relative quantitation method.

For the analysis of cytokine and chemokine gene expression in ILCs, the RT^2^ Profiler PCR Array kit for mouse cytokines and chemokines (PAMM-150ZA, Qiagen) was used with the ABI 7500 system. Heat map data were analyzed using the MeV software.

### Cytokine analysis

To measure the level of cytokines secreted from ILCs, lin^−^CD45^+^CD90.2^+^ cells prepared from PPs of BALB/c nude mice were pretreated with or without PT (500 ng/mL) for 6 hr, followed by butyrate treatment for a further 17 hr. The cells were then re-stimulated with PMA (50 ng/mL) and ionomycin (500 ng/mL). After 4 hr of stimulation, the culture medium was collected and the levels of cytokines were measured using the BD Cytometric Bead Array Mouse Th1/Th2/Th17 cytokine kit (BD Biosciences), according to the manufacturer’s protocol, and analyzed using the FCAP Array software. To measure the amount of IL-22, the Mouse/Rat IL-22 Quantikine ELISA kit (R&D Systems) was used according to the manufacturer’s protocol.

### Immunofluorescent staining

To monitor the expression of GPR109a, RORγt^gfp^ cells sorted from PPs of RORc(γt)^gfp^ reporter mice were fixed by 4% paraformaldehyde, pretreated with Mouse BD Fc Block, and stained with anti-GPR109a Ab, anti-NKp46 Ab, or anti-CCR6 Ab, followed by secondary Abs. The specimens were analyzed using confocal laser scanning microscopy (LSM 510 META, Carl Zeiss, Thornwood, NY). Data were analyzed using the LSM Image browser.

### Statistical analyses

Statistical analyses were performed using Prism 6 (GraphPad Software, La Jolla, CA). Data are presented as means ± SEs of repeated experiments, unless indicated otherwise. Differences between the means of multiple independent variables were compared between the control and treatment groups using one-way ANOVA followed by Tukey’s *post hoc* test. An unpaired Student’s *t*-test was used to compare two groups. Differences in mean values were considered significant at *p* < 0.05.

## Electronic supplementary material

Supplementary Information
