# Supplementary material for: Microbiota-derived butyrate suppresses group 3 innate lymphoid cells in terminal ileal Peyer’s patches
Source: Sci Rep. 2017 Jun 21;7:3980. doi: 10.1038/s41598-017-02729-6 (PMC5479798; doi:10.1038/s41598-017-02729-6)
Supplement: Supplementary file 1 — Supplementary Information [file 41598_2017_2729_MOESM1_ESM.pdf]

Supplementary information

Microbiota-derived butyrate suppresses group 3 innate lymphoid cells in terminal ileal Peyer's patches

Sae-Hae Kim[1,2], Byeol-Hee Cho[2], Hiroshi Kiyono[3], Yong-Suk Jang[1,2,*]

[1]Department of Molecular Biology and Institute for Molecular Biology and Genetics, Chonbuk National University, Jeonju 54896, Korea; [2]Department of Bioactive Material Sciences and Research Center of Bioactive Materials, Chonbuk National University, Jeonju 54896, Korea; [3]Division of Mucosal Immunology and International Research and Development Center for Mucosal Vaccines, Institute of Medical Science, The University of Tokyo, Tokyo 108-8639, Japan

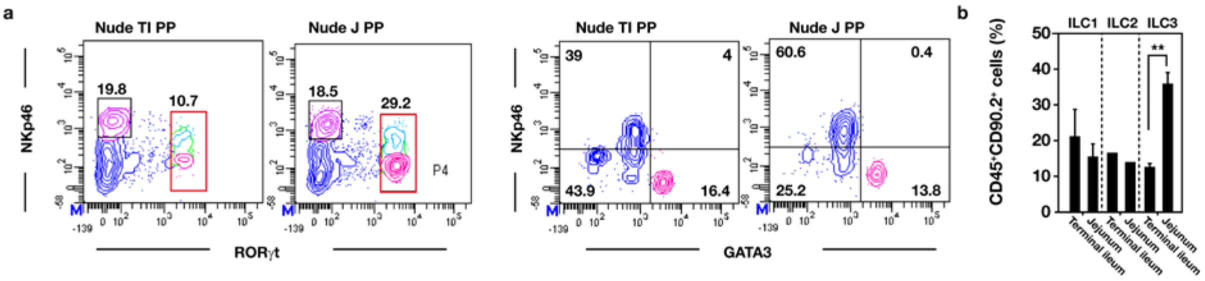

Supplementary Fig. 1. Frequency of ILC3s in PPs of BALB/c nude mice. (**a**) Distribution of ILCs in PPs of the terminal ileum (TI PP) and jejunum (J PP) of BALB/c nude mice was analyzed by flow cytometry according to the gating strategy explained in Fig. 1. The numbers shown indicate the percentage of cells in each gated area and data representative of three independent experiments are shown. (**b**) The numbers shown indicate the percentage of cells in each gated area and the data represent the mean ± SE calculated from three independent experiments with two mice per group. ***p* < 0.01 indicates a significant difference between the groups compared.

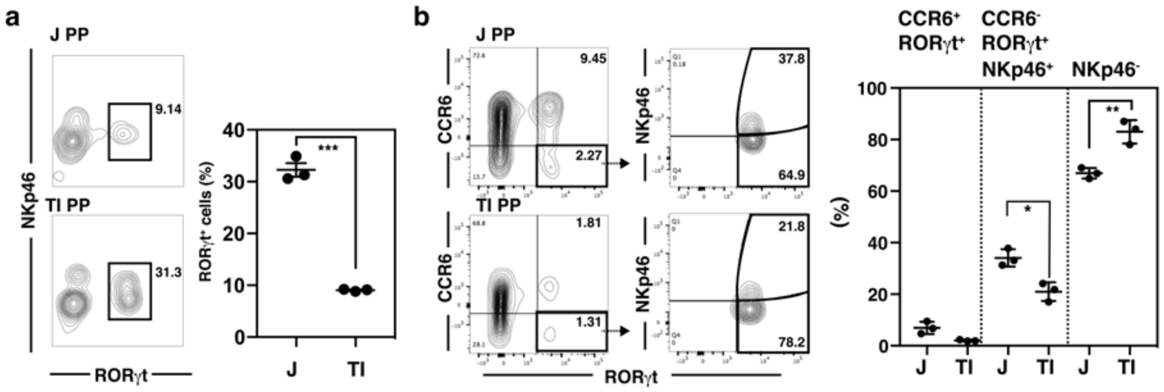

Supplementary Fig. 2. The RORγt^{+} ILC3s showed distinct distribution patterns between jejunal and terminal ileal PPs. (**a**) Distributions of ILC3s in PPs of terminal ileum (TI PP) and jejunum (J PP) of SPF mice without ABX treatment were analyzed by flow cytometry, according to the gating strategy explained in Fig. 1. (**b**) The frequencies of ILC3 subtypes (CCR6^{+}, CCR6^{-} NKp46^{+}, and CCR6^{-} NKp46^{-}) were analyzed in jejunal and terminal ileal PP cells of SPF mice without ABX treatment by flow cytometry. Data represent the mean ± SE calculated from three independent experiments with three mice per group. *$p < 0.05$, **$p < 0.01$, ***$p < 0.001$ indicate significant differences between the groups compared.

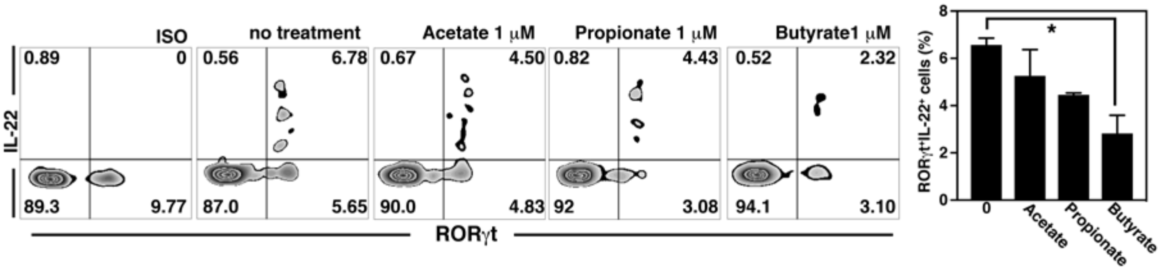

Supplementary Fig. 3. Butyrate could suppress IL-22 expression in RORγt$^+$ ILC3s. The jejunal PP cells had been stimulated *in vitro* with each indicated molecule and re-stimulated with PMA, ionomycin, and IL-23, and then the level of IL-22 in lin$^-$CD90.2$^+$CD45$^+$ RORγt$^+$ ILC3s was analyzed by flow cytometry. Data represent the mean ± SE calculated from three independent experiments with three mice per group. *$p < 0.05$ indicates a significant difference between the groups compared.

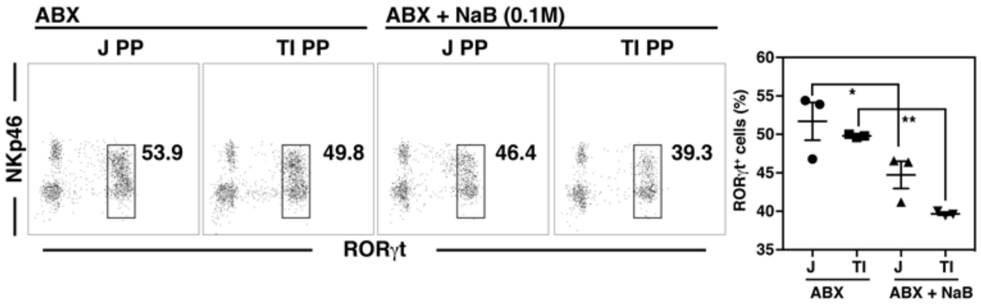

Supplementary Fig.4. Oral administration of NaB (0.1 M) can reduce the frequencies of RORγt$^+$ ILC3s in jejunal and terminal ileal PP cells of ABX-treated SPF mice. NaB was administered orally in ABX-treated SPF mice. After 17 hr, the frequency of lin$^-$CD90.2$^+$CD45$^+$RORγt$^+$ ILC3s in jejunal or terminal ileal PP cells was analyzed by flow cytometry. Data represent the mean ± SE calculated from three independent experiments with three mice per group. *$p < 0.05$, **$p < 0.01$ indicate significant differences between the groups compared.
